# Role of antibiotic envelopes in preventing cardiac implantable electronic device infection: A meta‐analysis of 14 859 procedures

**DOI:** 10.1002/joa3.12262

**Published:** 2019-11-18

**Authors:** Ashish Kumar, Rajkumar Doshi, Mariam Shariff

**Affiliations:** ^1^ Department of Critical Care Medicine St John's Medical College Hospital Bangalore India; ^2^ Department of Internal Medicine University of Nevada Reno School of Medicine Reno NV USA

**Keywords:** antibiotic envelope, cardiac implantable electronic devices

## Abstract

**Introduction:**

We conducted an updated meta‐analysis assessing the role of antibiotic envelopes in preventing Cardiac implantable electronic devices (CIED)‐related infections as compared to standard infection prevention strategies.

**Methods:**

A systematic search was conducted on Medline/PubMed and EMBASE/Ovid database. We used Mantel‐Haenszel method with fixed‐effect model to compute risk ratio (RR) with 95% confidence interval (CI). We also performed subgroup and trial sequential analysis on the data.

**Results:**

Antibiotic envelope reduced the risk of both all infections [RR: 0.41, CI: 0.31‐0.54, *P* < .05, *I* ^2^ = 75%, *χ* ^2^ *P* < .05] and major infections [RR: 0.48, CI: 0.32‐0.70, *P* < .05, *I* ^2^ = 60%, *χ* ^2^ *P* = .04].

**Conclusion:**

Prophylactic use of antibiotic envelopes as an adjuvant therapy to standard infection prevention strategies, helps in reducing the risk of CIED infections.

## Graphical abstract

Antibiotic envelope reduced the risk of both all infections and major infections. Prophylactic use of antibiotic envelopes as an adjuvant therapy to standard infection prevention strategies helps in reducing the risk of CIED infections.

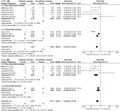

## 1 INTRODUCTION

Cardiac implantable electronic devices (CIED) play a role in ameliorating the quality of life and survival. With the increasing use of CIED, the related infection rates have also spiraled.^1^ Additionally, the associated morbidity and mortality following CIED infection is high.^2^ The use of prophylactic measures such as pre‐operative antibiotics to prevent CIED related infections is necessary. A recent and largest till date randomized controlled trial demonstrated that adjunctive use of antibacterial envelope is beneficial in terms of reducing periprocedural infection without added complication rates.^3^ Therefore, we conducted an updated meta‐analysis of observational studies and randomized control trials (RCTs) assessing the role of antibiotic envelopes in preventing CIED‐related infections as compared to standard infection prevention strategies.

## 2 METHODS

A systematic search was conducted on Medline/PubMed and EMBASE/Ovid database for relevant studies from inception to May 2019 using the following terms "Antibacterial envelope," "Antimicrobial envelope,” AIGISRx, TYRX, "TYRX‐A,” "Cardiac Implantable device infection,” "Infection." The online search was supplemented with a manual search of bibliographies from relevant articles and reviews. Two authors independently reviewed the title and abstract of searched citations to identify relevant articles and extracted data from included articles. The outcome of interest being infection rates. All infections and major infections were analyzed separately. Major infections were defined in all study as one of the following, infection requiring device removal, an invasive procedure for infection control, long term antibiotic followed by infection recurrence following discontinuation, or death. We used the Mantel‐Haenszel method with a fixed‐effect model to compute risk ratio (RR) with 95% confidence interval (CI). Heterogeneity was assessed using the *I* 2 test >60% or *χ* ^2^ *P* < .05. We also conducted a subgroup analysis for first‐generation/non‐absorbable antibiotic envelope and second‐generation/absorbable antibiotic envelope. Data were analyzed using RevMan Version 5.3 (Copenhagen: The Nordic Cochrane Centre, The Cochrane Collaboration, 2014). A trial sequential analysis adjusted confidence interval (TSA‐CI) for RR was calculated to accommodate for possible Type I error.

## 3 RESULTS

Database search yielded 66 studies, of which seven studies were included, six observational and one RCT, summing to a total of 14 859 procedures.^3^, ^4^, ^5^, ^6^, ^7^, ^8^, ^9^

**Figure 1:**
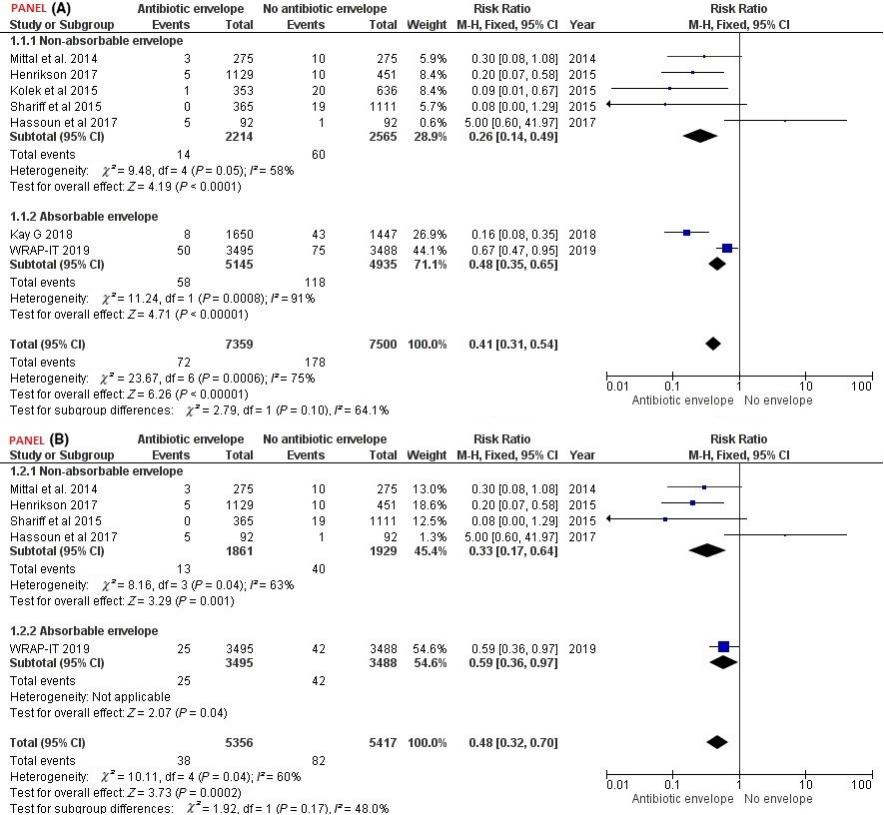
Panel A, Forest plot for risk of Infection (Major and/or minor infection). Panel B, Forest plot for risk of major infection. CI‐95%, Confidence interval, M‐H, Mantel‐Haenszel method

The use of antibiotic envelope was associated with significantly lower risk of CIED related infections (Major and/or minor) [RR: 0.41, CI: 0.31‐0.54, *P* < .05, *I* ^2^ = 75%, *χ* ^2^ *P* < .05]. The TSA‐CI for RR was also 0.14‐0.72. The TSA diversity adjusted information size calculated was 14 438. Both first and second‐generation antibiotic envelopes curtailed the risk of CIED related infection (Major and/or minor), first‐generation antibiotic envelope [RR: 0.26, CI: 0.14‐0.49, *P* < .05, *I* ^2^ = 58%, *χ* ^2^ *P* = .05], second‐generation antibiotic envelope [RR: 0.48, CI: 0.35‐0.65, *P* < .05, *I* ^2^ = 91%, *χ* ^2^ *P* < .05] (Figure 1 Panel A). Further, the use of antibiotic envelope was associated with significantly lower risk of CIED related major infection [RR: 0.48, CI: 0.32‐0.70, *P* < .05, *I* ^2^ = 60%, *χ* ^2^ *P* = .04] (Figure 1 Panel B). Both first‐ and second‐generation antibiotic envelopes curtailed the risk of CIED related major infection. Besides, high heterogeneity (as identified by *I* ^2^ and *χ* ^2^ *P*‐value) associated with all pooled estimates is to be considered during its interpretation.

## 4 DISCUSSION

The efficacy of antibiotic envelope in preventing CIED related infection was established in the present meta‐analysis which included 14 859 procedures, substantially expanding on the previous meta‐analysis which included 4779 procedures.^10^

Numerous studies have reported increased mortality and health cost expenditure associated with CIED infection.^2^ Until recently, there was limited evidence on effective prophylactic strategies to prevent infection during CIED procedures. Pre‐operative prophylactic (1 hour prior to surgery for cefazoline/ 2 hours prior to surgery for vancomycin) use of antibiotics with in‐vitro activity against staphylococci (cefazoline, vancomycin) is recommend as a class I indication (level of evidence: A) by the American heart association and heart rhythm society.^11^ Complete removal of the hardware is strongly recommended in patients with established CIED‐related infection. In 2008, the FDA approved the use of antibiotic envelope as a prophylactic strategy to prevent CIED infection. Several retrospective and observational studies have documented the efficacy of antibiotic envelopes in preventing CIED related infection. The WRAP‐IT trial, one of the most recent and largest till‐date, studied the efficacy and safety of antibiotic envelops as adjuvant therapy to standard infection prevention strategies. The study concluded that the use of antibiotic envelope resulted in a significantly lower incidence of major CIED infections as compared to standard of care infection prevention strategies.^3^ Our meta‐analysis was consistent with these findings and showed a reduced risk of CIED‐related infection (both major/ minor and, major only) with the use of antibiotic envelopes. Also, as interpreted from the TSA‐CI the possibility of Type I error associated with the result was snubbed and sufficient information size had been reached for the conclusion.

The use of an antibiotic envelope is associated with several deterrents. One must consider the cost associated with prophylactic antibiotic envelop use.^4^ Also, the effect of antibiotic envelops on hard clinical endpoints like death is not well established.^3^ Observational studies in the past failed to report an increased occurrence of post‐implantation mechanical complications like generator pocket hematoma, lead dislodgment and migration, etc in the antibiotic envelop cohort as compared to the non‐envelop cohort.^9^ However, these outcomes need further validation. Furthermore, though the present study, documented the beneficial effect of both first‐generation/non‐absorbable and second‐generation/absorbable envelope, the use of absorbable envelope provides an added benefit of no foreign body nidus for potential future infections.

The result of the meta‐analysis should be interpreted in light of several limitations. Most studies included in the analysis are not RCTs. The duration of follow up for each study has not been accounted for. Devices and antibiotics in each study were different. Lastly, the definition of infection varied among studies.

In conclusion, prophylactic use of antibiotic envelopes as an adjuvant therapy to standard infection prevention strategies, helps in reducing the risk of CIED infections.

## CONFLICT OF INTEREST

The authors declare no conflict of interests for this article.

## References

[1] Greenspon AJ , Patel JD , Lau E , Ochoa JA , Frisch DR , Ho RT , et al. 16‐year trends in the infection burden for pacemakers and implantable cardioverter‐defibrillators in the United States: 1993 to 2008. J Am Coll Cardiol. 2011;58(10):1001–6. 21867833 10.1016/j.jacc.2011.04.033

[2] Habib A , Le KY , Baddour LM , Friedman PA , Hayes DL , Lohse CM , et al. Predictors of mortality in patients with cardiovascular implantable electronic device infections. Am J Cardiol. 2013;111(6):874–9. 23276467 10.1016/j.amjcard.2012.11.052

[3] Tarakji KG , Mittal S , Kennergren C , Corey R , Poole JE , Schloss E , et al. Antibacterial envelope to prevent cardiac implantable device infection. N Engl J Med. 2019;380(20):1895–905. 30883056 10.1056/NEJMoa1901111

[4] Kay G , Eby EL , Brown B , Lyon J , Eggington S , Kumar G , et al. Cost‐effectiveness of TYRX absorbable antibacterial envelope for prevention of cardiovascular implantable electronic device infection. J Med Econ. 2018;21(3):294–300. 29171319 10.1080/13696998.2017.1409227

[5] Kolek MJ , Patel NJ , Clair WK , Whalen SP , Rottman JN , Kanagasundram A , et al. Efficacy of a bio‐absorbable antibacterial envelope to prevent cardiac implantable electronic device infections in high‐risk subjects. J Cardiovasc Electrophysiol. 2015;26(10):1111–6. 26222980 10.1111/jce.12768 PMC4607656

[6] Mittal S , Shaw RE , Michel K , Palekar R , Arshad A , Musat D , et al. Cardiac implantable electronic device infections: Incidence, risk factors, and the effect of the AigisRx antibacterial envelope. Hear Rhythm. 2014;11(4):595–601. 10.1016/j.hrthm.2013.12.013 24333543

[7] Hassoun A , Thottacherry ED , Raja M , Scully M , Azarbal A . Retrospective comparative analysis of cardiovascular implantable electronic device infections with and without the use of antibacterial envelopes. J Hosp Infect. 2017;95(3):286–91. 28131641 10.1016/j.jhin.2016.12.014

[8] Shariff N , Eby E , Adelstein E , Jain S , Shalaby A , Saba S , et al. Health and economic outcomes associated with use of an antimicrobial envelope as a standard of care for cardiac implantable electronic device implantation. J Cardiovasc Electrophysiol. 2015;26(7):783–9. 25845917 10.1111/jce.12684

[9] Henrikson CA , Sohail MR , Acosta H , Johnson EE , Rosenthal L , Pachulski R , et al. Antibacterial envelope is associated with low infection rates after implantable cardioverter‐defibrillator and cardiac resynchronization therapy device replacement: results of the citadel and centurion studies. JACC Clin Electrophysiol. 2017;3(10):1158–67. 29759500 10.1016/j.jacep.2017.02.016

[10] Ali S , Kanjwal Y , Bruhl SR , Alo M , Taleb M , Ali SS , et al. A meta‐analysis of antibacterial envelope use in prevention of cardiovascular implantable electronic device infection. Ther Adv Infect Dis. 2017;4(3):75–82. 28634537 10.1177/2049936117702317 PMC5467856

[11] Baddour LM , Epstein AE , Erickson CC , Knight BP , Levison ME , Lockhart PB , et al. Update on cardiovascular implantable electronic device infections and their management. Circulation. 2010;121(3):458–77. 20048212 10.1161/CIRCULATIONAHA.109.192665

